# 2363. Three-Month Safety and Immunogenicity of Bivalent SARS-CoV-2 Omicron-Containing Booster Vaccines: Interim Results From a Phase 3, Randomized, Observer-Blind, Active-Controlled Trial

**DOI:** 10.1093/ofid/ofad500.1984

**Published:** 2023-11-27

**Authors:** Ivan T Lee, Catherine A Cosgrove, Patrick Moore, Marcin Bula, Philip Kalra, Paul Dargan, Rebecca Clark, Marta Boffito, Ray Sheridan, Claire Bethune, Fiona Burns, Dinesh Saralaya, Rhiannon Nally, Christopher J A Duncan, David Chadwick, Adrian Palfreeman, Alberto San Francisco Ramos, Paul T Heath, Bethany Girard, Kristen Sellers, Elizabeth de Windt, Andrea Sutherland, LaRee Tracy, Honghong Zhou, Jacqueline Miller, Frances Priddy, Spyros Chalkias, Rituparna Das

**Affiliations:** Moderna, Inc., Cambridge, Massachusetts; Vaccine Institute, Centre for Neonatal and Paediatric Infection, St George’s University of London, London, England, United Kingdom; The Adam Practice and University Hospital Southampton NHS Foundation Trust, Southampton, England, United Kingdom; University of Liverpool, Liverpool, England, United Kingdom; Salford Royal Hospital and Northern Care Alliance NHS Foundation Trust, Salford, England, United Kingdom; Guy’s & St Thomas’ NHS Foundation Trust and King’s College London, London, England, United Kingdom; Layton Medical Centre, Blackpool, England, United Kingdom; Chelsea and Westminster Hospital NHS Foundation Trust and Imperial College London, London, England, United Kingdom; Royal Devon University Hospital, Exeter, England, United Kingdom; University Hospitals Plymouth NHS Trust, Plymouth, England, United Kingdom; University College London, London, England, United Kingdom; National Institute for Health Research Patient Recruitment Centre and Bradford Teaching Hospitals NHS Foundation Trust, Bradford, England, United Kingdom; Wansford and Kings Cliffe Practice, Wansford, England, United Kingdom; Translational and Clinical Research Institute, Newcastle University and NIHR Newcastle Clinical Research Facility, The Newcastle upon Tyne Hospitals NHS Foundation Trust, Newcastle upon Tyne, England, United Kingdom; South Tees Hospitals NHS Foundation Trust, James Cook University Hospital, Middlesbrough, England, United Kingdom; University Hospitals of Leicester NHS Trust, Leicester, England, United Kingdom; Vaccine Institute, Centre for Neonatal and Paediatric Infection, St George’s University of London, London, England, United Kingdom; Vaccine Institute, Centre for Neonatal and Paediatric Infection, St George’s University of London, London, England, United Kingdom; Moderna, Inc., Cambridge, Massachusetts; Moderna, Inc., Cambridge, Massachusetts; Moderna, Inc., Cambridge, Massachusetts; Moderna, Inc., Cambridge, Massachusetts; Moderna, Inc., Cambridge, Massachusetts; Moderna, Inc., Cambridge, Massachusetts; Moderna, Inc., Cambridge, Massachusetts; Moderna, Inc., Cambridge, Massachusetts; Moderna, Inc., Cambridge, Massachusetts; Moderna, Inc., Cambridge, Massachusetts

## Abstract

**Background:**

Updated COVID-19 vaccines have been developed to help broaden protection against circulating SARS-CoV-2 variants. Here we present a 3-month interim analysis from a phase 3, randomized, observer-blind, active-controlled study of 2 omicron BA.1-containing vaccines: BA.1-monovalent mRNA-1273.529 (BA.1 variant only) and BA.1-bivalent mRNA-1273.214 (SARS-CoV-2 Wuhan-Hu-1 strain and BA.1 variant).

**Methods:**

This multicenter study evaluated safety and immunogenicity of the BA.1-monovalent (Part 1) and -bivalent (Part 2) vaccines in individuals aged ≥ 16 years in the United Kingdom (NCT05249829). Eligible participants previously received 2 or 3 doses of an authorized/approved COVID-19 vaccine and were randomly assigned 1:1 to receive a 50-µg booster of the original mRNA-1273 vaccine or either BA.1-monovalent or -bivalent vaccines. Safety and reactogenicity up to 3 months post-booster were evaluated. Immunogenicity objectives were to demonstrate non-inferiority and/or superiority of BA.1-monovalent or -bivalent vaccine-elicited immune responses to BA.1 and the ancestral strain (D614G) at Day 29 and Month 3 compared with a booster dose of mRNA-1273 in participants without evidence of baseline SARS-CoV-2 infection up to day of analysis visit on Day 29 or Month 3.

**Results:**

At the interim analysis, 724 (Part 1) and 2824 (Part 2) booster recipients were included in the safety analysis set. BA.1-monovalent and -bivalent booster vaccines were well-tolerated with no new safety concerns identified. Compared with mRNA-1273, a fourth dose of BA.1-monovalent or -bivalent vaccine elicited superior nAb responses against omicron BA.1 at Month 3, with geometric mean ratios (GMRs) of 1.77 (96% CI, 1.55-2.02) and 1.67 (96% CI, 1.54-1.81), respectively. Non-inferior (lower bound of confidence interval ≥ 0.67) nAb responses against D614G were also demonstrated with GMRs of 0.80 (95% CI, 0.71-0.90) for BA.1-monovalent and 1.11 (96% CI, 1.03-1.18) for -bivalent vaccines.

**Conclusion:**

The BA.1 monovalent and bivalent booster vaccines elicited nAb responses against BA.1 that remain superior to mRNA-1273 at 3 months post-booster with no new safety concerns. These results support variant-updated vaccines to protect against evolving SARS-CoV-2.

**Disclosures:**

**Ivan T. Lee, MD, PhD**, Moderna, Inc.: Salary|Moderna, Inc.: Stocks/Bonds **Catherine A. Cosgrove, PhD**, Novavax: Advisor/Consultant|Novavax: Grant/Research Support|Novavax: Speaker **Patrick Moore, MRCGP**, AstraZeneca: Advisor/Consultant|AstraZeneca: Grant/Research Support|GSK: Advisor/Consultant|GSK: Grant/Research Support|Janssen: Advisor/Consultant|Janssen: Grant/Research Support|Medicago: Advisor/Consultant|Medicago: Grant/Research Support|Moderna, Inc.: Advisor/Consultant|Moderna, Inc.: Grant/Research Support|MSD: Advisor/Consultant|MSD: Grant/Research Support|Novavax: Advisor/Consultant|Novavax: Grant/Research Support|Sanofi: Advisor/Consultant|Sanofi: Grant/Research Support **Philip Kalra, MD**, Astellas: Advisor/Consultant|Astellas: Grant/Research Support|AstraZeneca: Advisor/Consultant|AstraZeneca: Grant/Research Support|Bayer: Advisor/Consultant|Bayer: Grant/Research Support|Evotec: Advisor/Consultant|Evotec: Grant/Research Support|Fresenius: Advisor/Consultant|Fresenius: Grant/Research Support|GSK: Advisor/Consultant|GSK: Grant/Research Support|Lilly: Advisor/Consultant|Lilly: Grant/Research Support|Otsuka: Advisor/Consultant|Otsuka: Grant/Research Support|Pfizer: Advisor/Consultant|Pfizer: Grant/Research Support|Pharmacosmos: Advisor/Consultant|Pharmacosmos: Grant/Research Support|Unicyte: Advisor/Consultant|Unicyte: Grant/Research Support|Vifor: Advisor/Consultant|Vifor: Grant/Research Support **Paul Dargan, MBBS**, Moderna, Inc.: Grant/Research Support **Marta Boffito, MD, PhD, FRCP**, AstraZeneca: Honoraria|ATEA: Advisor/Consultant|ATEA: Honoraria|Gilead: Advisor/Consultant|Gilead: Grant/Research Support|Gilead: Honoraria|GSK: Advisor/Consultant|GSK: Grant/Research Support|Moderna: Grant/Research Support|MSD: Advisor/Consultant|MSD: Grant/Research Support|MSD: Honoraria|Novavax: Grant/Research Support|Pfizer: Advisor/Consultant|Pfizer: Grant/Research Support|Roche: Advisor/Consultant|Roche: Grant/Research Support|Valneva: Grant/Research Support|ViiV: Advisor/Consultant|ViiV: Grant/Research Support **Fiona Burns, PhD**, Gilead Sciences Ltd.: Grant/Research Support|Gilead Sciences Ltd.: Speaker Fees **Christopher J. A. Duncan, DPhil**, MRC: Grant/Research Support|Wellcome Trust: Grant/Research Support **David Chadwick, PhD**, Gilead Sciences Ltd: Grant/Research Support|GSK: Grant/Research Support|Janssen: Grant/Research Support|Moderna, Inc.: Grant/Research Support|Novavax: Grant/Research Support|ViiV Healthcare: Grant/Research Support **Adrian Palfreeman, MBBS**, University Hospital of Leicester NHS Trust. - Leicester (United Kingdom): Employee **Paul T. Heath, FRCPCH**, AstraZeneca: Grant/Research Support|Janssen: Grant/Research Support|Moderna, Inc.: Grant/Research Support|Novavax: Grant/Research Support|Pfizer: Grant/Research Support|Valneva: Grant/Research Support **Bethany Girard, Ph.D.**, Moderna, Inc.: salary|Moderna, Inc.: Stocks/Bonds **Kristen Sellers, PhD**, Moderna, Inc.: Salary|Moderna, Inc.: Stocks/Bonds **Elizabeth de Windt, MPH**, Moderna, Inc.: Salary|Moderna, Inc.: Stocks/Bonds **Andrea Sutherland, M.D., MPH**, Moderna, Inc.: Salary|Moderna, Inc.: Stocks/Bonds **LaRee Tracy, PhD**, Moderna, Inc.: Salary|Moderna, Inc.: Stocks/Bonds **Honghong Zhou, Ph.D.**, Moderna, Inc.: salary|Moderna, Inc.: Stocks/Bonds **Jacqueline Miller, MD**, Moderna, Inc.: salary|Moderna, Inc.: Stocks/Bonds **Frances Priddy, MD, MPH**, Moderna, Inc.: Salary|Moderna, Inc.: Stocks/Bonds **Spyros Chalkias, MD**, Moderna, Inc.: Salary|Moderna, Inc.: Stocks/Bonds **Rituparna Das, M.D.**, Moderna, Inc.: Salary|Moderna, Inc.: Stocks/Bonds

